# Chitosan-Based Active Films Enriched with Thyme Essential Oil for Extending the Shelf Life of Refrigerated Chicken Meat

**DOI:** 10.3390/foods15183347

**Published:** 2026-09-21

**Authors:** Miriam Sánchez-Ordóñez, Irene Orts, Sara Martillanes, María del Rosario Ramírez-Bernabé, Jonathan Delgado-Adámez

**Affiliations:** Centro de Investigaciones Científicas y Tecnológicas de Extremadura (CICYTEX), Instituto Tecnológico Agroalimentario de Extremadura (INTAEX), Avda. Adolfo Suárez s/n, 06007 Badajoz, Spain

**Keywords:** biodegradable packaging, active packaging, thyme essential oil, chitosan, chicken breast, refrigerated

## Abstract

The development of biodegradable active packaging systems incorporating natural antimicrobial and antioxidant compounds represents a promising strategy for improving the quality and shelf life of fresh meat. This study aimed to develop chitosan (CH)-based active films containing thyme essential oil (TEO) at 0%, 2.5%, and 3%, and to evaluate their antimicrobial and antioxidant properties and effectiveness in preserving chicken breast fillets during refrigerated storage. The chemical composition of TEO was characterised by gas chromatography–mass spectrometry, identifying 29 compounds, with *p*-cymene (30.56%) and thymol (24.59%) as the predominant constituents. Undiluted TEO showed strong antimicrobial activity against *E. coli* and *L. innocua*. The incorporation of 2.5% and 3% TEO into CH films increased the inhibition percentage and microbial susceptibility and also enhanced their antioxidant activity. The performance of the films was subsequently assessed using chicken breast fillets stored at 5 °C for 1, 3, and 5 days. All CH-based systems reduced microbial growth compared with uncoated controls, which exceeded 6–7 log CFU g^−1^ for several microbial groups after 5 days. CH-based packaging also reduced protein oxidation and maintained low lipid oxidation. However, 3% TEO caused significant colour deterioration by day 5. These results demonstrate the potential of CH-based active films enriched with TEO as a natural and biodegradable packaging alternative to enhance the shelf life and safety of refrigerated chicken meat.

## 1. Introduction

The food industry faces significant challenges in moving towards more sustainable models. Within the European Union, packaging represents 36% of municipal solid waste, and it is one of the main consumers of virgin materials, accounting for 40% of plastics and 50% of paper used in Europe [1]. Among the main challenges are reducing the use of conventional plastics and replacing them with more environmentally friendly alternatives, reducing food waste, and improving food safety in an increasingly globalised and demanding market [2]. These challenges are closely interrelated and require solutions that not only minimise environmental impact but also ensure food quality, safety, and shelf life. International initiatives such as the 2030 Agenda for Sustainable Development promote reductions in food waste and more sustainable production systems. In Europe, Regulation (EU) 2025/40 [3] reinforces this transition by imposing stricter requirements on plastic packaging, recyclability, and the use of non-biobased materials. A sustainable transition requires combining advances in packaging, preservation methods, and resource efficiency within a circular-economy framework, considering the entire product life cycle. This integrated approach is essential to develop solutions that meet current food sector demands without compromising safety or long-term environmental balance. In this context, biodegradable biopolymers have emerged as promising candidates, as they offer functional characteristics comparable to traditional materials [4]. These biodegradable packaging materials act as protective barriers, inhibiting the growth of microorganisms, reducing oxidation, and delaying deterioration [5].

Thyme (*Thymus vulgaris*) essential oil (TEO) is widely recognised as a natural and safe source of bioactive phenolic compounds, mainly thymol and carvacrol, which are responsible for its pronounced antioxidant and antimicrobial properties [6,7,8]. These compounds have demonstrated strong inhibitory activity against both Gram-positive and Gram-negative foodborne pathogens, through mechanisms that primarily involve disruption of bacterial cell membranes and oxidative stress modulation [6,8,9]. From a technological perspective, the incorporation of essential oils such as TEO into biopolymeric matrices represents an attractive strategy for the development of active food packaging [7,10].

Chitosan (CH) is a type of degradable biopolymer of natural origin that is easy to obtain from agro-food industry waste, presenting itself as an environmentally and economically sustainable alternative to traditional single-use plastics [11]. CH is a linear polysaccharide consisting of 2-amino-deoxy-β-d-glucan (1,4)-linkage derived from the deacetylation of chitin, a component of the exoskeleton of crustaceans. Chitin is insoluble in water and is the second most abundant polysaccharide in nature, after cellulose [12]. The main characteristics that make CH suitable for food applications are its biodegradability, biocompatibility, non-toxicity, renewable nature, and excellent film-forming capacity. Although CH can form films without the addition of plasticizers, compounds such as glycerol are frequently incorporated to improve film flexibility, reduce brittleness, and enhance the mechanical and handling properties of the resulting materials [13].

Another interesting feature of CH is its high solubility in acidic solutions (mainly below pH = 6.0), being a weak base (pKa = 6.3) due to the presence of amine groups. At low pH values, the amine groups are positively charged due to protonation, so chitosan can be a water-soluble cationic polyelectrolyte [14]. In addition, CH exhibits antioxidant and antimicrobial activity against fungi, yeasts, and both Gram-positive and Gram-negative bacteria [15]. This activity is partly due to the CH interacting with the negative groups of the bacterial membrane, destabilizing it and causing its rupture [16]. The significant antimicrobial and antioxidant activity of CH has been widely demonstrated [17,18,19], making this biopolymer particularly attractive for the development of active food packaging materials. Furthermore, its combination with other substances, such as polysaccharides [20], proteins [21] or inorganic materials [22] has been studied. For instance, Mirsharifi et al. [23] reported that an almond gum/polyvinyl alcohol/chitosan matrix incorporating a TEO nanoemulsion, applied as an edible coating, effectively improved the microbiological quality and extended the shelf life of chicken breast fillets. In contrast, the present study evaluates chitosan-based films enriched with TEO as active packaging materials for refrigerated chicken meat, with special emphasis not only on microbial quality but also on lipid oxidation and colour stability, two key factors determining the shelf life and consumer acceptability of poultry meat [24].

The preservation of fresh meat, such as chicken, represents a significant challenge, as they are vulnerable to microbiological spoilage and physicochemical changes that compromise essential attributes such as colour, aroma, and flavour [25]. These characteristics are not only determinants for the perception of quality, but also for the purchasing decision and consumer satisfaction. During storage, meat is particularly susceptible to oxidation processes and microbial growth, which reduces its shelf life and negatively affects its quality. In this regard, packaging designed with protective films that act as physicochemical barriers has become a key solution to preserve the freshness of chicken fillets [26]. It prevents oxidation reactions and microbiological growth, thus prolonging shelf life and consumer appeal. Studies have reported that the combination of CH with TEO is a good option for preserving chicken fillets [27]. In addition, other authors have also demonstrated the beneficial effects of chitosan-based films enriched with essential oils in meat systems [28,29,30,31].

The main objective of this study is to develop an active CH biopolymer with TEO added at different levels to improve the preservation of chicken breast meat during refrigerated storage.

## 2. Materials and Methods

### 2.1. Essential Oil Extraction

The TEO (*Thymus vulgaris*) was provided by Biottonia Natural Care S.L. (Badajoz, Spain). The extraction of the thyme essential oil (TEO) was carried out by vapour distillation, at temperatures between 65 and 95 °C for 1–7 h (not exceeding 75 °C in the interior of the plant mass to avoid the loss of volatile compounds).

### 2.2. Chemical Composition Analysis of TEO

The chemical composition of TEO was determined using a Varian CP-3800 gas chromatograph equipped with a CombiPAL autosampler (CTC Analytics, Zwingen, Switzerland) and an HP-5 capillary column (30 m × 0.32 mm × 0.25 μm; Agilent Technologies, Santa Clara, CA, USA). For analysis, 10 μL of TEO was placed into a 20 mL vial, and a 1 cm DVB/CAR/PDMS SPME fiber (50/30 μm; Supelco, Bellefonte, PA, USA) was exposed to the vial headspace at 37 °C for 30 min. The injector temperature was set at 270 °C. The oven temperature program started at 35 °C (held for 10 min), increased to 250 °C at 7 °C/min, and was held for 5 min, resulting in a total runtime of 45 min. The transfer line, trap, and manifold temperatures were maintained at 280 °C, 200 °C, and 60 °C, respectively.

TEO compounds were identified using an Agilent 6850 Series Gas Chromatography/Agilent 5975 Series Quadropole Mass Detector (Single) (Agilent Technologies, Santa Clara, CA, USA). The MS source and MS Quad temperatures were maintained at 230 °C and 150 °C, respectively, scanning at 1 scan s^−1^ over a 40–300 *m*/*z* range. Identification was achieved by comparing mass spectra with the NIST library using Agilent MSD Chemstation E.02.01.1177 software and/or LRI reported in the literature [32,33,34]. TEO compound identification was confirmed by matching the linear retention indices and mass spectra with commercially acquired standards (Sigma-Aldrich, St. Louis, MO, USA). Samples were analyzed in triplicate, and the relative percentage of each compound in TEO was calculated from peak areas.

### 2.3. Preparation of Chitosan (CH) Active Biopolymers

For the present study, high-molecular-weight chitosan (CH) (Sigma-Aldrich, Madrid, Spain; 419419) was used as the film-forming biopolymer. To prepare the film-forming solutions, CH was dissolved in lactic acid, which promoted its solubilization through the protonation of amino groups and facilitated the formation of a homogeneous polymeric matrix [14]. Glycerol was subsequently incorporated as a plasticizer to improve film flexibility and reduce brittleness. The resulting solutions were homogenized in water under continuous stirring at 700 rpm for 30 min at 50 °C using an Agimatic-N stirrer (Selecta, Barcelona, Spain). Subsequently, thyme essential oil was incorporated, and the dispersion was homogenized at high speed to obtain a stable and uniform emulsion, ensuring an even distribution of the oil throughout the chitosan matrix. The concentrations of chitosan, lactic acid and glycerol were maintained constant based on previous studies from our research group in which these conditions produced films with appropriate physicochemical and technological properties [30,35,36]. Therefore, TEO concentration was selected as the only experimental variable.

Based on this formulation, three different chitosan-based films were prepared, as detailed in Table 1.

**Figure 1:**
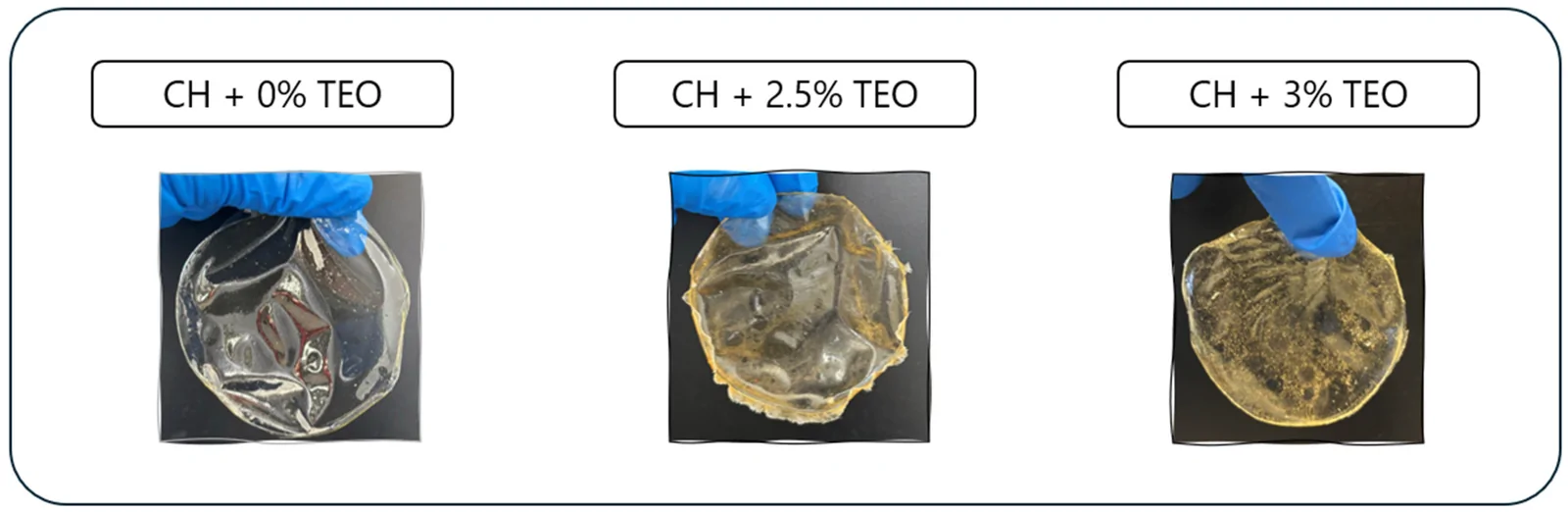
CH active biopolymers with different percentages of TEO.

All the biopolymers were weighed out to 20 g in a Ø 90 mm Petri dish. All were dried for 5 days at 22 °C in the dark until a flexible film was obtained (Figure 1). The doses of essential oil were chosen on the basis of the maximum level of essential oil that did not interfere with the polymerization process of the films. The activity of the developed biopolymers was evaluated in three independent films.

### 2.4. Antimicrobial Activity In Vitro

#### 2.4.1. Broth Microdilution in TEO

The antimicrobial activity and minimum inhibitory concentration (MIC) of TEO were determined by broth microdilution, in accordance with the spectrophotometric procedure described by Delgado-Adámez et al. [37]. *L. innocua* 911 and *E. coli* 45 were obtained from the Spanish Type Culture Collection. Bacterial strains were cultured for 24 h at 37 °C in Mueller–Hinton broth (MHB), and bacterial suspensions were adjusted to 0.5 McFarland. Each well received 190 µL of MHB containing approximately 10^5^ CFU mL^−1^ and 10 µL of TEO or its serial dilutions. The TEO was prepared at 0.1 g in 10 mL of water, followed by 1:10 and 1:100 aqueous dilutions.

A positive control (bacteria without extract) and a negative control (extract without bacteria) were included on each 96-well microplate. Each well (except negative control) contained an initial inoculum of approximately 10^5^ CFU mL^−1^. Since antimicrobial activity was assessed by spectrophotometric monitoring of bacterial growth, viable cell counts were not determined before or after incubation. After mixing, the plates were incubated at 37 °C for 24 h under aerobic conditions. Absorbance was recorded at 450 nm using a spectrophotometer (Thermo Scientific Electron APPLISKAN Type: 2001). This wavelength was selected after preliminary assays showed lower spectrophotometric interference from the intrinsic colour and optical properties of the essential oil compared with measurements at higher wavelengths, including 600 nm, thereby providing a more reliable estimation of bacterial growth inhibition. Similar approaches have been reported in antimicrobial studies involving coloured plant-derived extracts and essential oils, where the detection wavelength was selected to minimise background interference and improve the accuracy of spectrophotometric measurements [38,39].

All assays were performed in triplicate, and in vitro antimicrobial activity was expressed as % inhibition relative to the growth control. Antibacterial activity was calculated using the following Equation (1):

(1) $$\%\;\mathrm{Inhibition}=\frac{{\Delta \mathrm{Abs}}_{\mathrm{reference}}-\;{\Delta \mathrm{Abs}}_{\mathrm{assay}}}{{\Delta \mathrm{Abs}}_{\mathrm{reference}}}\;\times 100$$

Δ*Abs_reference_*: Increase in absorbance of the positive control sample (bacteria without extract).

Δ*Abs_assay_*: Increase in absorbance of the tested sample (bacterial growth in the presence of the extract).

#### 2.4.2. Agar Diffusion Assay of TEO and CH Biopolymers

The susceptibility of both TEO and CH-based biopolymers was evaluated using an agar diffusion assay adapted from the Kirby–Bauer method [40]. PCA plates were previously inoculated with the target microorganisms, *L. innocua* 910 and *E. coli* 45.

For the TEO assay, sterile paper discs (6 mm diameter) were impregnated with 30 µL of TEO diluted to 1% (*v*/*v*) in dimethyl sulfoxide (DMSO) to facilitate diffusion into the hydrophilic medium. Discs containing chloramphenicol (3 mg L^−1^ in ultrapure water) were used as positive controls. Chloramphenicol was selected as a reference broad-spectrum antimicrobial commonly employed in agar diffusion susceptibility assays to verify microorganism responsiveness and assay performance. Its use in the present study was intended exclusively as a methodological benchmark and was not related to any potential food application of the compound.

For the biopolymer assay, film samples were aseptically cut into 6 mm discs and placed directly on the inoculated agar surface.

All plates were incubated at 36 °C for 24 h, after which antimicrobial activity was quantified by measuring inhibition zones (mm) according to the National Committee for Clinical Laboratory Standards guidelines. All assays were performed in triplicate.

#### 2.4.3. Broth Macrodilution in CH Biopolymers

The bacteriostatic activity of the CH biopolymers was evaluated using a broth macrodilution assay. Cultures of *L. innocua* 911 and *E. coli* 45 were reactivated in MHB and incubated at 37 °C for 18–20 h. Bacterial suspensions were adjusted to 10^6^ cells mL^−1^ using a Neubauer chamber, ensuring a final inoculum of approximately 10^4^ cells mL^−1^ in the assay tubes.

CH biopolymers were aseptically cut into 10 × 15 mm pieces. Each biopolymer fragment was placed in 50 mL Falcon tubes containing 10 mL MHB. Two experimental conditions were evaluated: a positive control containing only MHB and the bacterial inoculum, and a test condition consisting of MHB, the bacterial inoculum and the CH biopolymer. All tubes were inoculated with 100 µL of the standardized bacterial suspension and incubated at 37 °C for 18–20 h under shaking.

After incubation, suitable dilutions of each culture were pour-plated (1 mL) on PCA and incubated at 37 °C for 24 h to determine final microbial counts (CFU mL^−1^), which were compared with the positive controls to assess bacteriostatic activity. Results were expressed as % inhibition relative to the growth of each pathogen, using the following Equation (2):

(2) $$\%\;\mathrm{Inhibition}=\frac{N_{\mathrm{reference}}-\;N_{\mathrm{assay}}}{N_{\mathrm{reference}}}\;\times 100$$

N_reference_: Microbial count (CFU mL^−1^) of the positive control.

N_assay_: Microbial count (CFU mL^−1^) of the sample containing the CH biopolymer.

Negative values obtained when Na > Nr were considered indicative of no inhibition and were therefore recorded as 0% for statistical analysis.

### 2.5. Antioxidant Activity (ABTS Method)

Antioxidant activity was assessed by monitoring the absorbance at 734 nm, corresponding to the scavenging of the ABTS•^+^ radical. The assay was performed following the method of Turoli et al. [41], using a multimode microplate reader (Tecan Spark, Grödig, Austria).

For TEO, an ethanolic solution was obtained by mixing 10 µL of essential oil with 990 µL of ethanol, and 50 µL of this solution was then combined with 1 mL of ABTS to initiate the reaction. Regarding CH biopolymers, small circles (6 mm Ø) were placed in 1 mL of ABTS to initiate the reaction. Antioxidant activity was performed in triplicate and expressed as mM Trolox for TEO and as mmol eq. Trolox (cm^2^)^−1^ for the different CH biopolymers.

### 2.6. Chicken Fillets Preparation and Packaging Method

For the evaluation of the effect on meat, 30 biopolymers of the three types were prepared for each experiment. Two independent experiments were carried out: one for the analysis of the microbiological effect and another for the analysis of the physical-chemical effect on meat quality (colour and oxidation parameters).

Fresh chicken breast fillets of approximately 10 g were obtained from a local market (Badajoz, Spain) on the day of processing and transported to the laboratory in insulated ice boxes within 30 min. Fillets were selected to ensure similar size, weight and appearance, and all samples used in each experiment were obtained from the same batch to minimize biological variation and kept at 5 ± 1 °C before packaging.

The control samples were vacuum-packaged in polyamide/polyethylene bags (20/100, 50 cm^3^ m^−2^, 24 h^−1^, and 0% relative humidity, 120 µm thickness, Eurobag, S.L., Alicante, Spain) using a vacuum packaging machine (HenkoVac, Busch, Germany) without biopolymer. For the remaining treatments, CH active biopolymers (CH + 0% TEO, CH + 2.5% TEO, or CH + 3% TEO) were placed on both sides of the central area of each chicken fillet conforming to the shape of the package, and then vacuum packed in polyamide/polyethylene bags (20/100, 50 cm^3^ m^−2^, 24 h^−1^, and 0% relative humidity, 120 µm thickness, Eurobag) using a vacuum packaging machine (HenkoVac, Busch, Germany). The CH-based films were used as active inserts within a vacuum-packaged multilayer system rather than as stand-alone packaging materials. This approach was selected because biopolymer films commonly exhibit limited moisture and gas barrier performance compared with conventional plastic packaging, whereas multilayer systems allow the active functionality of the biopolymer to be combined with the protective properties of commercial vacuum packaging.

Samples were stored in a temperature-controlled refrigerator (Balay^®^, Zaragoza, Spain) at 5 ± 1 °C, corresponding to the refrigeration conditions commonly used for fresh poultry meat during commercial distribution and domestic storage. Storage was carried out for 1 (T1), 3 (T3), and 5 (T5) days, and temperature fluctuations were maintained within this range throughout the experimental period.

After the storage time, the colour parameters and microbiological analysis were immediately measured. The rest of the samples were frozen until analysis.

Five replicates were analysed for each experimental batch; a total of 60 fillets were analysed in each experiment: 4 types of packaging × 3 times of storage × 5 replicates.

### 2.7. Breast Chicken Composition

Analysis was carried out on 5 breast fillets. Moisture was determined according to the AOAC (2016) [42], protein content was determined from the nitrogen content (N × 6.25) by the Kjeldahl method [43] and fat content was analysed by Folch et al. [44] method. Results were expressed as g 100 g^−1^ sample.

The fatty acid profile was determined by gas chromatography (GC), analysing the fatty acid methyl esters (FAMEs) obtained from the methylation of the fat extract with KOH in methanol with an Agilent Technologies 6890 GC (Agilent Technologies, Santa Clara, CA, USA) equipped with a flame ionization detector and an Agilent DB-23 (60 m × 0.25 mm ID × 0.25 μm) capillary column. A 1 μL aliquot was injected in split mode (1:100). Split liners with glass wool and 11 mm septa were used. The injector and detector temperatures were maintained at 260 °C and 280 °C, respectively. The oven temperature program started at 185 °C (held for 2 min), then increased at 5 °C min^−1^ to 220 °C and held for 15 min. Helium was used as a carrier gas at a constant flow of 1.2 mL min^−1^. Fatty acids were identified by comparison of their retention times with those of a commercial FAME Mix 37 Component standard (Supelco, LRAD1445, Bellefonte, PA, USA) analyzed under the same chromatographic conditions. Results were expressed as a percentage of the total identified fatty acids.

### 2.8. Microbiological Analyses of Chicken Breast

For microbiological analyses, 10 g of sample were aseptically collected from the whole fillet, including both surface and inner portions, to obtain a representative sample. Microbiological analyses were performed according to international standards. Counts of mesophilic and psychrophilic aerobes [45], total coliforms [46], *E. coli* [47], and *Staphylococcus aureus* [48] were evaluated. The analyses were carried out under the cultivation conditions indicated for each determination. Ten grams of meat were diluted in a decimal series until the appropriate dilution was achieved for the loads of each microorganism in each sample. Results were expressed as log CFU g^−1^.

### 2.9. Instrumental Colour of Chicken Breast

Colour was measured instrumentally by assessing the parameters of the CIELAB scale. Lightness (CIE L*), redness (CIE a*), and yellowness (CIE b*) were obtained with a Konica Minolta CM-5 spectrophotometer (Konica Minolta, Tokyo, Japan) with 30 mm aperture, illuminance D65, and a viewing angle of 10°. The measurements were performed directly on the fillet surface on both sides of each fillet.

### 2.10. Oxidation Status of Chicken Breast

Prior to the determination of lipid and protein oxidation parameters, chicken fillets were chopped and homogenized using a food grinder to obtain a representative sample. The homogenized meat was then used for the corresponding analytical determinations.

Lipid oxidation was evaluated using thiobarbituric acid reactive substances (TBA-RS) based on the method described by Sorensen & Jorgensen [49]. Briefly, an amount of 4 g of previously chopped sample was weighed and homogenised with 15 mL of TCA (prepared with 1 g propylgallate + 1 g EDTA + 75 g trichloro acetic acid in 1 L of distilled water) for 45 s. Then, 4 mL of the previously filtered homogenate was mixed with 4 mL of TBA (thiobarbituric acid, 20 mM) and heated for 40 min at 90 °C. After this time, the samples were cooled (10–15 min) to room temperature. These samples were centrifuged for 10 min at 2500 rpm, and the absorbance of the supernatant was read at 508, 532, and 600 nm using a UV-Visible spectrophotometer (Thermo Scientific Evolution 201, Madrid, Spain). TBARS values were calculated from a standard curve of tetraethoxypropane (TEP) and expressed as milligrams of malondialdehyde (MDA) per kilogram of sample (mg MDA kg^−1^).

Protein oxidation was assessed by estimation of carbonyl groups formed during incubation with 2,4-dinitrophenylhydrazine (DNPH) in 2 N HCl following the method described by Oliver et al. [50]. For this, 1 g of sample was homogenised with 10 mL of 0.15 M KCl for 1 min, and two different 100 μL aliquots were taken and 1 mL of 10% TCA (trichloroacetic acid) was added to them. After shaking and centrifugation (5 min, 10,000 rpm), the supernatants were removed and 1 mL of 2 N HCl and 1 mL of 2 N HCl + 0.2% DNPH, respectively, were added to the residues obtained. After 1 h of reaction, 1 mL TCA was added to each aliquot and the supernatant was removed by centrifugation. The pellet thus obtained was washed with a mixture of ethanol: ethyl acetate (1:1) three times and finally redissolved in 6 M guanidine solution. Carbonyl concentration was evaluated by measuring DNPH incorporated based on the absorption of 21 mM 1 cm 1 at 370 nm for protein hydrazones. Protein concentration was calculated by spectrophotometry at 280 nm using a UV-Visible spectrophotometer (Thermo Scientific Evolution 201, Madrid, Spain). Results were expressed in nmol carbonyls mg protein^−1^.

### 2.11. Statistical Analysis

Data were analyzed using the statistical software package SPSS version 21.0 (SPSS Inc., Chicago, IL, USA). All results are shown as mean ± standard deviation for each designed group. One-way analysis of variance (ANOVA) (*p* ≤ 0.05) was applied to evaluate the effects of bacterial species (*E. coli*, *L. innocua*), antimicrobial agent, antioxidant activity in the CH biopolymer formulation, and to compare the effect of biofilms in chicken breast fillets. Tukey’s test was applied to compare the mean values when ANOVA showed significant differences.

## 3. Results and Discussion

### 3.1. Chemical Composition of TEO

Table 2 shows the chemical composition of TEO (*T. vulgaris*) after analysis by gas chromatography. The chemical analysis revealed the presence of 29 identified compounds, with *p*-cymene (30.56%) and thymol (24.59%) as the major constituents, collectively accounting for about 55% of the total TEO composition. Norouzi et al. [51] reported thymol followed by *p*-cymene and γ-terpinene among the major constituents of TEO. For instance, Wang et al. [52] identified thymol as the predominant compound in *T. vulgaris* oil, with higher concentrations likely associated with the geographical origin of collection. Variations in the relative abundance of these compounds among studies may be attributed to differences in geographical origin of the plant material, climatic and edaphic conditions, genetic variability among chemotypes, agronomic practices, harvesting stage, and extraction methodology [51,53].

Compounds such as *p*-cymene and thymol have demonstrated potent antimicrobial activity due to their ability to disrupt the integrity of the bacterial cell membrane, causing the loss of ions and other components essential for cell viability [54,55,56]. The remaining compounds were present in proportions ranging from 8.35% to 0.02%. Although less abundant, they are not less relevant, since essential oils are rich in phenolic and terpenoid constituents such as α-pinene, β-pinene, and carvacrol, compounds associated with antioxidant and antimicrobial activities [6,57]. In addition, minor constituents may enhance the antimicrobial activity of essential oils through synergistic interactions among different components [58].

### 3.2. Antimicrobial Activity and Minimum Inhibitory Concentration of TEO

The antimicrobial activity of TEO, evaluated by a broth microdilution method to determine the minimum inhibitory concentration (MIC), is summarised in Table 3.

In relation to the comparison in inhibition percentage between microorganisms for each TEO concentration, no significant differences were observed between *E. coli* and *L. innocua* when treated with undiluted TEO or with the 1:10 dilution. In contrast, significant differences were detected at the lowest concentration tested (1:100), where *L. innocua* exhibited a higher inhibition percentage (71.86%) than *E. coli* (65.17%).

With respect to the comparison between TEO concentrations within each microorganism, significant differences in inhibition percentage were observed for both *E. coli* and *L. innocua*. In both cases, undiluted TEO and the 1:10 dilution showed the highest inhibition percentages, whereas a significant reduction in antimicrobial activity was detected at the 1:100 dilution. These results indicate that the antimicrobial activity of TEO is clearly concentration-dependent, with the highest efficacy achieved in its pure form or at moderate dilution levels. As extract concentration decreases, the availability of bioactive compounds is reduced, leading to a progressive decline in microbial inhibition.

The MIC for both *E. coli* and *L. innocua* was reached with undiluted TEO, showing inhibition values of 92.39% and 95.38%, respectively. These results reflect the high antimicrobial potency of TEO, mainly associated with its major bioactive compounds, such as thymol, carvacrol, and *p*-cymene, which act by disrupting the bacterial cell membrane and inhibiting microbial growth at sufficiently high concentrations [8,9,55].

This dose-dependent behaviour is consistent with previous reports. Aguado et al. [59] observed substantial antimicrobial effects of TEO in its pure form and when diluted to 50% in water against *Listeria monocytogenes*, *E. coli*, and *Salmonella typhimurium*. Similarly, Acosta et al. [60] reported that *Thymus vulgaris* essential oil diluted at 1:10 and 1:20 retained antimicrobial activity against several strains of *Escherichia coli* and *Staphylococcus aureus*, reinforcing the relevance of moderate dilutions in preserving antimicrobial efficacy.

### 3.3. Susceptibility by Agar Diffusion in TEO

The bactericidal activity, as measured by agar diffusion (mm halo), is reported in Table 3. It analyses the susceptibility of microorganisms (*E. coli* and *L. innocua*) to each antimicrobial agent and the differences between antimicrobial agents within each microorganism.

In this assay, chloramphenicol was selected as a control due to its well-established antimicrobial efficacy and broad-spectrum activity [61], allowing the effectiveness of TEO to be benchmarked against a reference antibiotic. Moreover, TEO was evaluated in its undiluted form, as previous results demonstrated that the MIC reached under these conditions provided the most effective bactericidal response.

First, chloramphenicol shows higher susceptibility to *E. coli* (36.76 mm halo) than *L. innocua* (30.76 mm halo). The differences in susceptibility of *E. coli* and *L. innocua* may be due to differences in the structure and composition of their lipid membrane [62]. In contrast, TEO exhibited no significant difference in susceptibility between the two microorganisms studied. This uniform response may be attributed to the complex mixture of bioactive phytochemicals present in TEO, such as carvacrol, thymol, and *p*-cymene, which act synergistically on different parts of the bacterial cell [54]. TEO also exhibits strong antimicrobial capacity, a feature consistently supported by previous studies: Drioiche et al. [63] reported potent antimicrobial activity of TEO against Gram-negative bacteria, attributed to the effect of phenols. Moreover, Aljabeili et al. [6] observed that TEO showed significant antimicrobial activity, generating a broad inhibition zone against *Listeria monocytogenes*.

Regarding the comparative analysis between the different antimicrobial agents studied (chloramphenicol and TEO) shows significant differences in their effectiveness against the same microorganism. In the case of *E. coli*, chloramphenicol shows a higher susceptibility, with a halo of 36.76 mm, compared to TEO (29.86 mm halo). Similar trends were observed when the different antimicrobial agents were evaluated against *L. innocua*. This effect is expected given that chloramphenicol is a small, highly diffusive broad-spectrum antibiotic that inhibits protein synthesis, resulting in strong antimicrobial activity even at low concentrations [61]. In contrast, TEO consists of hydrophobic phytochemicals such as carvacrol, thymol, and *p*-cymene, whose limited diffusion in hydrophilic agar and membrane-targeted mode of action typically yield smaller halos despite their high antimicrobial potency [54]. Furthermore, the outer membrane of Gram-negative bacteria constitutes an additional permeability barrier that restricts the diffusion of hydrophobic compounds [62].

### 3.4. Antimicrobial Activity of CH Biopolymers

For the food to be shielded from microbial development and kept fresh for a long time, the active food packaging sheet must have strong antimicrobial activity [64]. Table 4 summarises the antimicrobial activity (% inhibition) obtained by the macrodilution assay, highlighting the influence of both the target microorganism and the percentage of TEO incorporated into the CH biopolymer.

With respect to the comparison between microorganisms (*E. coli* and *L. innocua*) for the same formulation, CH + 0% TEO and CH + 3% TEO exhibited no significant inhibitory effects against both microorganisms. In contrast, CH + 2.5% TEO showed significantly higher growth inhibition against *E. coli* than against *L. innocua*, for which no bacteriostatic effect was detected. This result suggests that, although the formulation with 2.5% TEO contains antimicrobial compounds, their availability or persistence over time was insufficient to exert a sustained inhibitory effect against *L. innocua* under broth conditions. This behaviour is consistent with the antimicrobial mode of action of major TEO constituents, such as thymol and *p*-cymene, which primarily induce rapid membrane disruption rather than prolonged bacteriostatic inhibition [54].

Regarding the comparison between CH biopolymer formulations within the same microorganism, clear concentration-dependent effects were observed. For *E. coli*, antimicrobial inhibition increased progressively with the percentage of TEO incorporated into the film, reaching the highest inhibition level at 3% TEO. A similar trend was observed for *L. innocua*, for which CH + 3% TEO showed the strongest bacteriostatic effect. The higher antimicrobial efficacy observed at increased TEO concentrations in CH biopolymers can be attributed to the greater availability of bioactive compounds, mainly thymol, whose membrane-disruptive action is strongly concentration-dependent and enhanced by synergistic components such as *p*-cymene [54].

Some studies have shown that CH films with TEO have different antimicrobial activity depending on the percentage of essential oil used. Guzmán-Pincheira et al. [65] showed that, *L. innocua* was susceptible to essential oils incorporated into alginate-chitosan films, with inhibitory effects observed at low concentrations.. Ballester-Costa et al. [66] demonstrated that CH films with essential oils from different varieties of thyme were more effective against *L. innocua* when they contained 2% essential oil compared to those formulated with 1%. Ruiz-Navajas et al. [10] reported that a higher content of thyme oil in CH films increased the inhibition halo against *L. innocua*.

### 3.5. Susceptibility by Agar Diffusion CH Biopolymers

Table 4 presents the susceptibility expressed as inhibition halos (mm), determined by the disc diffusion assay, which evaluates the bactericidal activity of CH biopolymers incorporating different percentages of TEO.

When susceptibility was compared between microorganisms within the same formulation, no statistically significant differences were observed. Films containing 0%, 2.5%, or 3% TEO produced similar responses in *E. coli* and *L. innocua*, indicating that the bactericidal action of the CH–TEO systems was comparable for both bacterial species under agar diffusion conditions. This absence of differential susceptibility between microorganisms is noteworthy, as Gram-negative bacteria are typically considered more resistant due to the presence of an outer membrane enriched in lipopolysaccharides, which can limit the access of active compounds to the cytoplasmic membrane [67,68].

However, a different trend emerged when each microorganism was evaluated across the various CH formulations. In this case, marked differences were detected among the films. CH + 2.5% TEO generated the largest inhibition areas against both bacteria, whereas CH + 3% TEO showed an intermediate inhibition area effect. By contrast, films without essential oil (CH + 0% TEO) exhibited the weakest antibacterial response. This behaviour is consistent with the intrinsic properties of CH-based films, whose antimicrobial activity under agar diffusion conditions is mainly restricted to direct contact due to limited polymer diffusion [69]. The incorporation of TEO enhanced bacterial susceptibility, with CH acting not only as a structural matrix but also as a carrier that promotes the availability of essential-oil constituents at the material–bacteria interface, thereby facilitating membrane-level interactions that are effective against both Gram-positive and Gram-negative microorganisms [54]. In line with this, Elsebaie et al. [69] demonstrated that CH films produced measurable inhibition halos only after the incorporation of green pea pod extracts, confirming that the addition of bioactive extracts enhances antimicrobial activity under agar diffusion conditions.

### 3.6. Antioxidant Activity of TEO and CH Biopolymers

As shown in Table 5, TEO exhibited 86.068 mM Trolox antioxidant capacity, confirming its strong radical-scavenging potential. This pronounced activity is mainly associated with its high content of thymol and *p*-cymene. Thymol, as a phenolic compound, contributes directly to antioxidant activity through its ability to donate hydrogen atoms and interrupt oxidative chain reactions [70,71], whereas *p*-cymene, although showing limited antioxidant action on its own, plays a synergistic role by enhancing the effectiveness of thymol and stabilising the overall antioxidant system [6,7,8].

Regarding the antioxidant performance of the CH biopolymers, significant differences were observed among the formulations. CH films without essential oil (CH + 0% TEO) showed lower antioxidant activity, while the incorporation of TEO markedly enhanced the antioxidant capacity of the biopolymers. Both CH + 2.5% TEO and CH + 3% TEO presented significantly higher antioxidant values. Antioxidant capacity increased with essential oil due to the presence of phenolic terpene compounds in TEO [7]. The incorporation of natural extracts and essential oils has been widely reported to enhance the antioxidant activity of CH-based films [72,73]. For instance, Hao et al. [29] observed improved DPPH radical scavenging activity in CH films enriched with oregano essential oil and black rice bran anthocyanins. However, Cabeza de Vaca et al. [30] reported a decrease in antioxidant activity in CH films containing rice bran extract after 6 and 24 h, attributed to the dispersion of the extract within the polymer matrix.

### 3.7. Breast Chicken Composition

As the elemental composition and fatty acid profile were determined solely for the purpose of establishing the initial characteristics of the chicken fillets prior to their preservation using CH films with TEO, these results are presented descriptively in the text rather than in a specific table.

The moisture content was 76.47 ± 0.92 g 100 g^−1^, protein content 23.04 ± 0.70 g 100 g^−1^, and total fat content 1.28 ± 0.27 g 100 g^−1^. Regarding fatty acid composition: Palmitic acid (C16:0) was the major saturated fatty acid (24.87 ± 0.75%), oleic acid (C18:1) was the predominant monounsaturated fatty acid (38.49 ± 0.92%), and linoleic acid (C18:2, cis–cis) was the main polyunsaturated fatty acid (20.98 ± 0.40%). The ∑ saturated, ∑ monounsaturated, and ∑ polyunsaturated fatty acids accounted for 33.86 ± 1.59%, 44.00 ± 1.03%, and 22.52 ± 0.53%, respectively. The chemical composition and fatty acids of the chicken fillets fell within the ranges reported in previous studies [74].

These compositional characteristics contribute to the highly perishable nature of chicken meat. Its high moisture content provides a plentiful supply of water, whilst its high protein content supplies nutrients that promote the growth of pathogenic and spoilage-causing microorganisms [75,76]. Consequently, chicken breast is considered a product with a limited shelf life, even when stored under refrigerated conditions [77]. Furthermore, the high proportion of unsaturated fatty acids increases the susceptibility of chicken meat to lipid oxidation and resulting off-flavors [24].

### 3.8. Effect of CH Films with TEO for Fresh Chicken Preservation

#### 3.8.1. Microbiology

The analysis of microbiological counts in chicken breast packaged with different biopolymers is shown in Table 6.

At T1 (day 1 of refrigeration), microbial counts of mesophilic and psychrophilic bacteria, total coliforms, and *E. coli*. were highest in the control samples, which had no biopolymer coating. In contrast, all coated samples (CH + 0% TEO, CH + 2.5% TEO, and CH + 3% TEO) showed reduced levels. Specifically, in *E. coli* counts, the most notable effect was observed in the active biopolymers CH + 2.5% TEO and CH + 3% TEO, below the detection limit (<1 log CFU g^−1^). For *S. aureus*, only the CH + 2.5% TEO treatment achieved full inhibition at T1, with counts below the detection limit (<10 log CFU g^−1^). This result indicates a potent antimicrobial effect arising from the synergistic action of CH and TEO, both recognized for their ability to disrupt bacterial cell membranes and inhibit essential metabolic processes [15,78,79].

After 3 days of storage (T3), mesophilic counts were highest in the control and CH + 3% TEO samples. For psychrophilic bacteria and total coliforms, the highest counts were again found in the control group, while CH + 0% TEO and CH + 2.5% TEO treatments significantly reduced microbial loads. The CH + 3% TEO packaging maintained intermediate levels for these microbial groups, suggesting a slightly lower efficacy during mid-storage. *S. aureus* remained below the detection limit (<10 log CFU g^−1^) in all treated samples.

After 5 days of refrigerated storage (T5), chicken breast fillets without active packaging (control samples) exhibited mesophilic, psychrophilic, total coliform, and *E. coli* counts exceeding 6–7 log CFU g^−1^, which is considered the threshold at which sensory deterioration becomes evident according to Commission Regulation (EC) No 1441/2007 [80]. The high microbial counts observed in the control samples at day 5 indicate that the product had reached the end of its microbiological shelf life under the storage conditions evaluated. This extensive microbial proliferation was considerably reduced in samples packaged with chitosan-based films, demonstrating their ability to delay microbial spoilage during refrigerated storage. However, the active biopolymers significantly reduce the growth of these microorganisms. *S. aureus*, as in the T3 treatment, remained below the detection limit (<10 CFU g^−1^) in all chicken fillet samples stored in the different types of packaging.

The present results confirm that active CH biopolymer coatings, particularly those enriched with TEO, are effective in limiting the proliferation of microorganisms in chicken fillets during refrigerated storage. The antimicrobial efficacy was attributed to phenolic and terpenoid compounds in TEO, particularly thymol, carvacrol, and γ-terpinene, which exert their action by disrupting bacterial membranes and increasing cell permeability, mechanisms that may underlie the strong inhibitory effects observed in our active packaging [81,82]. Similar antimicrobial effects have been documented in prior studies. For example, Zheng et al. [26] reported that incorporating oregano essential oil into CH coatings at concentrations ranging from 0.25% to 2.0% significantly improved their antimicrobial efficacy in total viable count. Similarly, Abdalbeygi et al. [83] observed a marked inhibition of *E. coli* O157:H7 in chicken meat coated with chitosan and essential oil formulations. Furthermore, Mehdizadeh & Mojaddar Langroodi [84] documented that chitosan coatings combined with natural bioactive compounds, such as propolis extract or Zataria multiflora essential oil, effectively suppressed spoilage bacteria throughout refrigeration. Mirsharifi et al. [27] demonstrated that active films containing TEO maintained microbial counts in meat below acceptable thresholds even after 21 days of cold storage.

#### 3.8.2. Colour

The colour parameters (L*, a*, and b*) of chicken breast packed with different active biopolymer systems are presented in Table 7. These values are essential indicators of visual freshness and strongly influence consumer acceptance.

At T1, lightness (L*) was highest in the control, CH + 0%, and CH + 2.5% TEO samples, while CH + 3% TEO showed significantly lower values, indicating a darker surface appearance. Redness (a*) did not differ significantly. For yellowness (b*), CH + 3% TEO showed the highest value, whereas the control exhibited the lowest. Although thyme essential oil and the active films possess an intrinsic yellowish colour, a direct colouring effect on the chicken fillets is considered unlikely due to the low concentration of essential oil incorporated into the films and the limited transfer of pigments from the packaging material to the meat. At T3 (day 3 of refrigerated conservation), differences in lightness were found. The control and CH + 2.5% TEO maintained high values, while CH + 3% TEO was significantly lower. However, the a* and b* values did not differ significantly at this stage. By day 5 (T5), a more evident degradation of color was observed in CH + 3% TEO samples, with significant reductions in L* and a* parameters. Whereas b* showed lower values in control and CH + 3% TEO samples.

**Figure 2:**
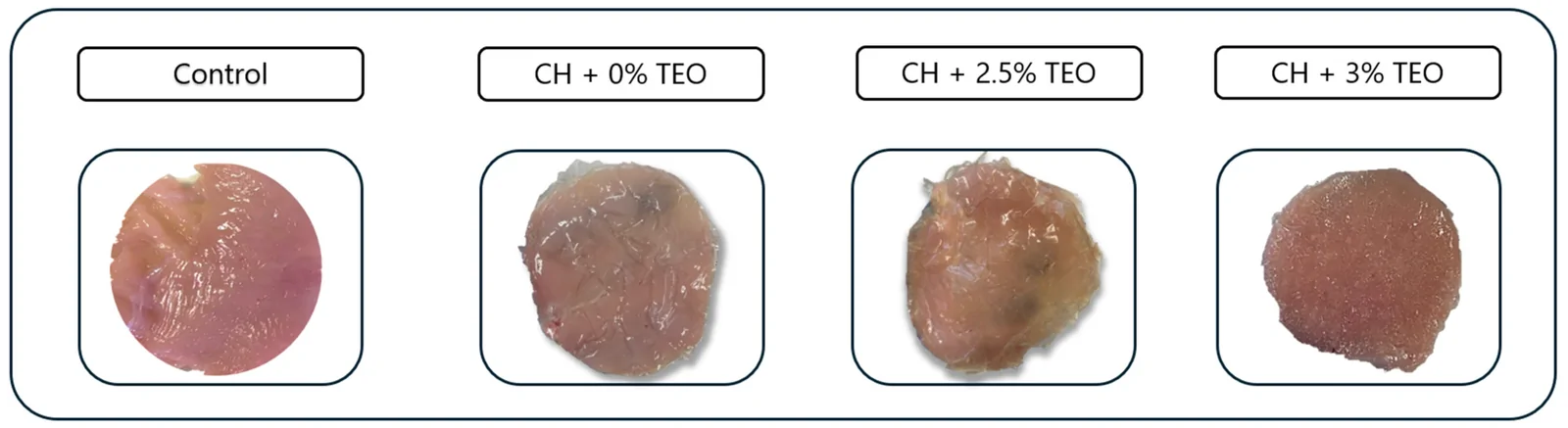
Representative appearance of chicken breast fillets after 5 days of refrigerated storage (T5) under the different packaging treatments (Control, CH + 0% TEO, CH + 2.5% TEO and CH + 3% TEO).

These findings suggest that, although TEO has strong antimicrobial properties, its concentration in biopolymer coatings must be carefully optimised to balance microbial inhibition and visual quality preservation. Although CH + TEO active packaging effectively maintained L*, a*, and b* values during storage, likely due to the combined antibacterial and antioxidant activities of CH, the formulation containing 3% TEO induced noticeable changes in color. In early storage (T1 and T3), this higher concentration mainly reduced lightness (L*), contributing to a darker appearance. By T5, the effect became more pronounced, exhibiting a visibly drier and rougher surface than the remaining treatments (Figure 2) with significant reductions in L*, a*, and b* values, suggesting that the biopolymer may have promoted moisture migration at the meat surface and progressive discoloration. This deterioration may be attributed to pigment interactions promoted by the phenolic structure and hydrophobic nature of TEO components such as thymol and carvacrol, which can alter the surface chromatic profile of the meat [7]. Specifically, the decline in a* values reflects the oxidation of oxymyoglobin to brown metmyoglobin (MetMb), a common indicator of color degradation in meat during refrigeration [85].

Previous studies have reported that CH packaging systems can improve colour stability in meat, with the effect being dependent on both the concentration of the extract and the storage duration. Zheng et al. [26] found that CH combined with oregano essential oil significantly delayed the discoloration of chicken breasts over 12 days of storage. They attributed these effects to the antioxidant and antimicrobial properties of both CH and the essential oil, which help protect myoglobin from oxidation and maintain surface integrity. Similarly, Wang et al. [86] demonstrated that nanoemulsion coatings with cinnamon essential oil effectively preserved colour parameters in chicken meat stored at 4 °C. In line with these results, Cardoso et al. [17] showed that coatings based on gelatin and CH enhance color stability in beef steaks, yielding a darker and more intense red appearance during display, even without altering myoglobin redox forms. Also, Cabeza de Vaca et al. [30] showed that gelatin/CH and CH films with rice bran extract prevented the color changes in meat during storage. Moreover, studies using CH-alginate films enriched with active nanocompounds have also reported improved color retention in red meat during 7 days of refrigerated storage [87].

#### 3.8.3. Changes in Oxidation Status

The lipid oxidation (measured as TBARS, mg MDA kg^−1^) and protein oxidation (nmols carbonyls mg protein^−1^) of chicken breast samples packed in different active biopolymer systems are shown in Table 8.

At T1, both TBARS and protein oxidation showed no significant differences among treatments, showing a lower initial oxidative status. Regarding T3, differences emerged. In TBARS, the control sample showed the lowest value, CH + 0% TEO, and CH + 2.5% TEO showed intermediate values, and CH + 3% TEO exhibited the highest oxidation. For protein oxidation, significant differences were observed: the control had the highest value, while CH + 0%, CH + 2.5%, and CH + 3% TEO active packaging all recorded lower values, indicating that each biopolymer coating, regardless of TEO concentration, reduced protein oxidation at T3. At T5, the lowest TBARS values were observed in the control and CH + 2.5% TEO, while the chicken breast samples coated with CH + 3% TEO exhibited intermediate levels, and CH + 0% TEO presented the highest values. For protein oxidation, no significant differences were detected, suggesting that by day 5, the coatings had a similar effect on protein carbonyl formation.

These results reveal that the oxidative stability of chicken breast was affected by both the biopolymer coating and the concentration of TEO. Notably, TBARS values did not follow a linear trend: at day 3, the lowest oxidation levels were detected in the control, while CH + 3% TEO exhibited the highest values; however, by day 5, the lowest TBARS values were observed in the control and in CH + 2.5% TEO, whereas CH without TEO showed the highest lipid oxidation levels. This may indicate a pro-oxidant effect of high TEO levels under certain conditions. It is important to note that, although no regulatory limits exist for TBARS values in meat, levels above 0.5 mg MDA/kg are generally associated with sensory changes such as rancidity, while values exceeding 1.0 mg MDA/kg may lead to consumer rejection [88]. In this study, all values remained within acceptable sensory tolerances on the fifth day. Furthermore, the consistently lower protein oxidation observed in coated samples suggests that the barrier properties and active components of the coatings (CH and TEO) were effective in protecting muscle proteins throughout the 1 to 5 days of refrigerated storage. Such coatings help limit oxidative damage by reducing oxygen exposure and delivering antioxidant compounds, which is crucial since protein oxidation alters protein structure and impairs functional properties [89].

These findings are further supported by Zheng et al. [26], who reported that CH coatings, especially when combined with oregano essential oil, significantly reduced TBARS values in chicken meat during refrigeration. Similarly, Yeddes et al. [31] demonstrated that gelatin–CH films enriched with Tunisian rosemary extract effectively reduced lipid oxidation in freeze-dried chicken meat during long-term storage. The antioxidant activity of chitosan is attributed to its hydroxyl and amino groups [90], and its coating can effectively reduce lipid oxidation by limiting oxygen diffusion [91]. Moreover, films containing TEO significantly delayed oxidative deterioration in meat, attributing this effect to phenolic components like thymol, carvacrol, and γ-terpinene, which act through dual antimicrobial and antioxidant mechanisms [27]. On the other hand, Jridi et al. [92] reported a significant reduction in TBARS values in beef coated with gelatin films enriched with *Lawsonia inermis* (henna) extract, highlighting the protective role of active biopolymer coatings in mitigating lipid oxidation during storage.

## 4. Conclusions

The present study demonstrates that chitosan-based biodegradable films enriched with thyme essential oil (TEO) constitute an effective form of active packaging capable of improving the preservation of chicken breast during refrigerated storage. The chemical profile of TEO, dominated by *p*-cymene and thymol, supports its strong antimicrobial and antioxidant potential, which was successfully transferred to the CH matrix. The minimum inhibitory concentration (MIC) for both *E. coli* and *L. innocua* was reached with undiluted TEO, highlighting the strong intrinsic bioactivity of the extract prior to its incorporation into the films.

Incorporating TEO into the films markedly enhanced their functional bioactivity, with the 2.5% and 3% formulations showing the most consistent antimicrobial inhibition against *E. coli* and *L. innocua*, as well as higher antioxidant activity. When applied to chicken breast fillets, CH–TEO films effectively reduced microbial growth, limited lipid and protein oxidation, and contributed to better colour stability compared with uncoated samples.

These findings position CH–TEO active films as a promising natural and biodegradable packaging alternative capable of extending shelf life and enhancing the quality and microbiological safety of refrigerated chicken meat. Nevertheless, further studies should include a comprehensive physicochemical characterization of the developed films, including mechanical, barrier, and structural properties, to better understand the relationship between film performance and preservation efficacy. Additionally, scale-up studies and sensory validation are needed to support their industrial application.

## Figures and Tables

**Table 1:** Preparation of different formulations of CH active biopolymers and physicochemical characteristics of the resulting films.

Formulation	Components	Thickness (mm)	Weight (mg)
CH + 0% TEO	CH 2% (*w*/*v*) Lactic acid 1% (*v*/*v*) Glycerol 1% (*w*/*v*)	0.036 ± 0.006	13.6 ± 0.5
CH + 2.5% TEO	CH 2% (*w*/*v*) Lactic acid 1% (*v*/*v*) TEO 2.5% (*w*/*v*) Glycerol 1% (*w*/*v*)	0.043 ± 0.013	15.2 ± 1.7
CH + 3% TEO	CH 2% (*w*/*v*) Lactic acid 1% (*v*/*v*) TEO 3% (*w*/*v*) Glycerol 1% (*w*/*v*)	0.042 ± 0.012	14.5 ± 1.9

CH: chitosan; TEO: Thyme essential oil. All biopolymers were dissolved in water.

**Table 2:** Chemical composition (%) of TEO.

No.	Compounds	Percentage (%)
1	*p*-Cymene	30.56 ± 1.5
2	Thymol	24.59 ± 0.2
3	Caryophyllene	8.35 ± 0.1
4	γ-Terpireno	7.59 ± 0.3
5	Isoborneol	4.48 ± 0.4
6	β-Pinene	3.94 ± 0.3
7	α-Terpinyl acetate	3.06 ± 0.2
8	β-Linalool	2.90 ± 0.9
9	Camphene	2.17 ± 0.2
10	α-Pinene	2.15 ± 0.2
11	Borneol	1.95 ± 0.4
12	Carvacrol	1.90 ± 0.1
13	Eucalyptol	1.45 ± 0.0
14	δ-Cadinene	0.92 ± 0.1
15	γ-Cadinene	0.76 ± 0.1
16	α-Humulene	0.57 ± 0.1
17	Alcanfor	0.48 ± 0.1
18	γ-Muurolene	0.40 ± 0.0
19	Caryophyllene oxide	0.40 ± 0.1
20	(-)-β-Pinene	0.36 ± 0.1
21	α-Copaene	0.25 ± 0.0
22	β-Bourbonene	0.24 ± 0.1
23	Isobornyl propionate	0.19 ± 0.0
24	Alloaromadendren	0.11 ± 0.0
25	β-Cuvebene	0.08 ± 0.0
26	α-Muurolene	0.07 ± 0.0
27	α-Calacorene	0.05 ± 0.0
28	Cedrol	0.03 ± 0.0
29	Cubenol	0.02 ± 0.0

TEO: Thyme essential oil.

**Table 3:** Antimicrobial activity (% inhibition) and susceptibility (mm halo) of TEO.

Antimicrobial Activity (% Inhibition)		
Antimicrobial Agent	*E. coli*	*L. innocua*
TEO (undiluted)	92.39 Aa ± 4.01	95.38 Aa ± 1.83
TEO (1:10)	86.93 Aa ± 1.25	88.10 Aa ± 2.90
TEO (1:100)	65.17 Bb ± 1.28	71.86 Ab ± 2.94
Susceptibility (mm halo)		
Antimicrobial agent	*E. coli*	*L. innocua*
Chloramphenicol (Control)	36.76 Aa ± 1.60	30.76 Ba ± 0.70
TEO (undiluted)	29.86 Ab ± 1.80	26.10 Ab ± 1.30

Values represent means ± standard deviation. Chloramphenicol was applied at a concentration of 3 mg L^−1^. TEO: Thyme essential oil. Uppercase letters indicate the statistical differences between microorganisms for each antimicrobial agent (comparison across columns). Lowercase letters indicate the statistical differences between antimicrobial agents within each microorganism (comparison across rows).

**Table 4:** Antimicrobial activity (% inhibition) and susceptibility (mm halo) of CH biopolymers with different percentages of TEO (0%, 2.5%, and 3%).

Antimicrobial Activity (% Inhibition)		
Antimicrobial Agent	*E. coli*	*L. innocua*
CH + 0% TEO	1.00 Ac ± 0.02	1.04 Ab ± 0.03
CH + 2.5% TEO	8.93 Ab ± 1.93	<0 Bc
CH + 3% TEO	17.84 Aa ± 0.47	19.98 Aa ± 0.72
Susceptibility (mm halo)		
Antimicrobial agent	*E. coli*	*L. innocua*
CH + 0% TEO	1.02 Ac ± 0.03	1.01 Ac ± 0.02
CH + 2.5% TEO	13.51 Aa ± 3.20	13.50 Aa ± 1.50
CH + 3% TEO	10.90 Ab ± 0.49	12.00 Ab ± 1.17

Values represent means ± standard deviation. CH: Chitosan. TEO: Thyme essential oil. <0: No inhibition. Different uppercase letters indicate the statistical differences between microorganisms for each antimicrobial agent (comparison across columns) (*p* ≤ 0.05). Different lowercase letters indicate the statistical differences between antimicrobial agents within each microorganism (comparison across rows) (*p* ≤ 0.05).

**Table 5:** Antioxidant activity of TEO (mM Trolox) and CH biopolymers with different percentages of TEO (mmol eq. Trolox (cm^2^)^−1^).

Antioxidant Activity	
TEO	86.068 ± 12.356
Biopolymers	
CH + 0% TEO	0.005 b ± 0.0003
CH + 2.5% TEO	0.046 a ± 0.0028
CH + 3% TEO	0.045 a ± 0.0023

Values represent means ± standard deviation. CH: Chitosan. TEO: Thyme essential oil. Different letters within the same column indicate statistical differences between different CH biopolymers (*p* ≤ 0.05).

**Table 6:** Microbiological counts (log CFU g^−1^) in chicken breast packed in different active biopolymer systems and stored under refrigeration for 1 (T1), 3 (T3), and 5 days (T5).

Microorganisms	Conservation Time	Packaging			
		Control	CH+	CH+	CH+
			0% TEO	2.5% TEO	3% TEO
Mesophilic	T1	4.53 a ± 0.21	3.70 b ± 0.20	3.16 b ± 0.54	3.74 b ± 0.12
Psychrophilic		4.49 a ± 0.25	3.34 b ± 0.24	3.38 b ± 0.09	3.61 b ± 0.09
Total coliforms		2.72 a ± 0.38	1.43 b ± 0.42	1.30 b ± 0.08	1.15 b ± 0.15
*E. Coli*		1.36 a ± 0.08	1.00 b ± 0.09	ND c	ND c
*S. aureus*		2.56 a ± 0.36	3.00 a ± 1.00	ND b	2.30 a ± 0.09
Mesophilic	T3	5.61 a ± 0.27	4.70 b ± 0.17	3.90 b ± 0.95	5.02 a ± 0.74
Psychrophilic		5.86 a ± 0.64	4.20 b ± 0.52	4.07 b ± 0.69	4.66 ab ± 1.01
Total coliforms		3.35 a ± 0.43	2.28 b ± 0.60	2.08 b ± 0.35	2.88 ab ± 0.42
*E. Coli*		2.52 a ± 0.48	1.30 b ± 0.30	1.65 ab ± 0.65	1.70 ab ± 0.09
*S. aureus*		ND a	ND a	ND a	ND a
Mesophilic	T5	>7 a ± -	6.11 b ± 0.72	6.91 b ± 0.76	6.25 b ± 0.28
Psychrophilic		>7 a ± -	6.41 b ± 0.70	6.63 b ± 0.43	6.60 b ± 0.32
Total coliforms		>6 a ± -	4.59 b ± 0.49	4.52 b ± 0.56	4.60 b ± 0.08
*E. Coli*		>6 a ± -	2.48 b ± 0.09	ND c	2.00 b ± 0.05
*S. aureus*		ND a	ND a	ND a	ND a

Values represent means ± standard deviation. CH: Chitosan. TEO: Thyme essential oil. Different letters within the same row indicate significant differences between different packaging (*p* ≤ 0.05). ND: Below the detection limit of the method ≤ 1 CFU g^−1^ and for *S. aureus* ≤ 10 CFU g^−1^.

**Table 7:** Colour in chicken breast packed in different active biopolymer systems and stored under refrigeration for 1 (T1), 3 (T3) and 5 days (T5).

Colour Parameters	Conservation Time	Packaging			
		Control	CH+	CH+	CH+
			0% TEO	2.5% TEO	3% TEO
L*	T1	55.2 a ± 1.2	55.2 a ± 2.3	55.4 a ± 1.2	51.2 b ± 2.0
a*		4.0 a ± 0.8	2.5 a ± 1.3	2.1 a ± 1.1	3.1 a ± 1.1
b*		10.6 b ± 2.1	11.8 ab ± 1.2	12.7 ab ± 0.5	13.5 a ± 1.4
L*	T3	58.6 a ± 0.9	53.8 ab ± 5.2	55.3 a ± 2.7	48.6 b ± 3.6
a*		3.9 a ± 1.2	1.3 a ± 1.5	2.9 a ± 1.3	2.8 a ± 1.7
b*		12.4 a ± 0.2	11.4 a ± 1.5	12.0 a ± 0.9	12.1 a ± 1.5
L*	T5	56.8 a ± 0.7	53.8 a ± 2.8	55.9 a ± 1.6	47.8 b ± 2.1
a*		3.6 a ± 1.1	3.0 a ± 0.8	2.2 ab ± 1.0	1.4 b ± 0.6
b*		11.7 b ± 0.7	13.8 a ± 0.9	13.1 ab ± 0.9	11.4 b ± 1.7

Values represent means ± standard deviation. CH: Chitosan. TEO: Thyme essential oil. Different letters within the same row indicate significant differences between different packaging (*p* ≤ 0.05).

**Table 8:** Lipid (TBARS, mg MDA kg^−1^) and protein oxidation (nmols carbonyls mg protein^−1^) in chicken breast packed in different active biopolymer systems and stored under refrigeration for 1 (T1), 3 (T3) and 5 days (T5).

Oxidation Parameters	Conservation Time	Packaging			
		Control	CH + 0% TEO	CH + 2.5% TEO	CH + 3% TEO
TBARS	T1	0.01 a ± 0.01	0.00 a ± 0.01	0.00 a ± 0.01	0.00 a ± 0.00
Protein oxidation		1.90 a ± 0.40	1.80 a ± 0.20	2.00 a ± 0.30	2.00 a ± 0.10
TBARS	T3	0.00 b ± 0.01	0.00 ab ± 0.01	0.01 ab ± 0.01	0.02 a ± 0.02
Protein oxidation		2.42 a ± 0.55	1.74 b ± 0.16	1.76 b ± 0.20	1.86 b ± 0.35
TBARS	T5	0.00 b ± 0.00	0.02 a ± 0.01	0.00 b ± 0.00	0.01 ab ± 0.01
Protein oxidation		1.76 a ± 0.32	1.74 a ± 0.25	2.04 a ± 0.23	1.94 a ± 0.09

Values represent means ± standard deviation. CH: Chitosan. TEO: Thyme essential oil. Different letters within the same row indicate significant differences between different packaging (*p* ≤ 0.05).

## Data Availability

The data presented in this study are available on request from the corresponding author due to what reason. The data are not publicly available because they form part of an ongoing research project and may be used in future publications.

## Abbreviations

The following abbreviations are used in this manuscript:

CH	Chitosan
TEO	Thyme essential oil
DMSO	Dimethyl sulfoxide
MHB	Mueller–Hinton broth
FAMES	Fatty acid methyl esters
FIND	Flame ionization detector
DNPH	2,4-dinitrophenylhydrazine
TBA-RS	Thiobarbituric acid
MDA	Malondialdehyde
TEP	1,1,1,3-tetraethoxypropane
ANOVA	Analysis of variance
MIC	Minimum inhibitory concentration
